# Time-Efficient Allocation Mechanisms for Crowdsensing Tasks with Precedence Constraints †

**DOI:** 10.3390/s19112456

**Published:** 2019-05-29

**Authors:** Xiaocan Wu, Yu-E Sun, He Huang, Yang Du, Danlei Huang

**Affiliations:** 1 The School of Computer Science and Technology, Soochow University, Suzhou 215006, China; 20184227017@stu.suda.edu.cn (X.W.); huangh@suda.edu.cn (H.H.); 20185227048@stu.suda.edu.cn (D.H.); 2 The School of Rail Transportation, Soochow University, Suzhou 215131, China; 3 The Suzhou Institute for Advanced Study, University of Science and Technology of China, Suzhou 215123, China; jannr@mail.ustc.edu.cn

**Keywords:** crowdsensing, task allocation, execution time minimization, precedence constraint, UAV

## Abstract

Crowdsensing has emerged as an efficient and inexpensive way to perform specialized tasks by leveraging external crowds. In some crowdsensing systems, different tasks may have different requirements, and there may be precedence constraints among them, such as the Unmanned Aerial Vehicle (UAV) crowdsensing systems. Moreover, minimizing the total execution time is a regular target for finishing the crowdsensing tasks with precedence constraints. As far as we know, only a few existing studies consider the precedence constraints among crowdsensing tasks, and none of them can minimize the total execution time simultaneously. To tackle this challenge, an efficient allocation mechanism for tasks with precedence constraints is first proposed, which can minimize the total execution time. Then, a case study is given to show how to fit our mechanism in the UAV crowdsensing system. Finally, the simulation results show that the proposed mechanisms have good approximate optimal ratios under different parameter settings and are efficient for the UAV crowdsensing system as well.

## 1. Introduction

With the emergence of various wireless technologies (4/5G, DSRC, and etc.), ubiquitous terminal equipment, such as smartphones, vehicles and UAVs, can collect real-time data from the environment and transmit the data to the IoT central server effectively [1,2,3]. As an important application of IoT, crowdsensing can leverage the power of large crowds to complete the complicated sensing tasks by using their smartphones or other mobile devices [4,5]. Compared with the conventional data collection methods, crowdsensing provides a low-cost and time-efficient solution for large-scale sensing tasks. With the dramatic proliferation of mobile devices, a set of crowdsensing systems have been implemented in recent year [6,7,8,9,10,11,12,13,14,15,16,17]. For instance, Kumar Rana et al. implemented an Ear-Phone system for monitoring the environmental noise pollution in urban areas through crowdsensing data collection [18].

Task allocation mechanism is crucial for crowdsensing, which directly decides the performance of the crowdsensing system. A variety of task allocation mechanisms have been proposed for crowdsensing systems [19,20,21,22,23,24,25,26,27,28,29,30,31]. For example, Reddy et al. proposed to maximize the spatial coverage with limited resource [19]. Jaimes et al. designed a budget-constrained incentive mechanism for task allocation [20]. He et al. took travel time into consideration and proposed to maximize the spatial coverage [21]. Considering that crowdsensing tasks may have various requirements (such as the type of data, sensing periods, etc.) and workers have different skills and reliability levels. Li et al. proposed to dynamically select appropriate workers for given tasks while keeping the constraints satisfied [22], Jin et al. incorporated quality of data to design the incentive mechanisms for MCS systems [25]. Iijima et al. considered the individual preference in distributed environments and proposed an adaptive task allocation mechanism that maximizes the social utility [32].

However, all these studies assume that the tasks in the crowdsensing system can be performed simultaneously and ignore that the sensing tasks may have precedence constraints in some applications. A crowdsensing task with precedence constraints cannot be executed before its pre-order tasks are finished. Actually, many crowdsensing applications have multiple steps, which will cause the precedence constraints of sensing tasks. UAV crowdsensing system is a typical system with precedence constraints, where exist mainly six types of tasks: WASD (Wide Area Search and Destroy), ISR (Intelligence Surveillance and Reconnaissance), CAS (Close Air Support), SEAD (Suppression of Enemy Air Defense), AR (aerial refueling), and PS (precision strike) [33,34]. In this system, there exists an execution sequence among tasks. Another example is the MCS based traffic congestion monitoring system, which monitors the traffic condition through collecting the sensing data of vehicles on different major roads. When a traffic jam occurs, the system will publish tasks to find out the reason that causes this congestion, to monitor the progress of the events that cause traffic congestion or to verify the effectiveness of the traffic grooming strategy. Obviously, these tasks have precedence constraints, i.e., the system needs to find out the congestion reason before monitoring the progress of the events.

Designing an efficient task allocation mechanism for tasks with precedence constraints meets more challenge than the existing ones. First, time efficiency, i.e., the total execution time of all the tasks, is usually important for these crowdsensing systems. Generally, task requesters want the total execution time as short as possible, such as in the UAV system [35,36,37,38]. However, different tasks may have different requirements for users, and each user can only meet the requirements of some tasks. The platform can only allocate tasks to the user who meets their requirements. Since all the constraints are taken into account, it is a hard job to allocate the tasks to users optimally, especially for the case users arrive online. As far as we know, only a few studies [39,40,41,42,43,44] have considered the precedence constraints among different tasks. For example, Schwarzrock et al. proposed a task allocation mechanism for UAV system, which can increase the amount of performed tasks [45]. However, none of these studies can minimize the total execution time of all the tasks at the same time.

To address this challenge, the crowdsensing task allocation problem with precedence constraints is studied in this paper, and an efficient allocation mechanism with the goal of minimizing the total execution time of tasks is designed. The NP-hardness of the studied problem can be proved by reducing the problem studied in this work to a classic NP-hard problem of multiprocessor scheduling problem (the details are as shown in Section 2.3), which means the studied problem does not exist a polynomial-time algorithm to get the optimal solution. Therefore, a near-optimal allocation mechanism is proposed to solve it. The designed mechanism includes four steps, which are task level division, final task set construction, allocation priority sequence construction, and task allocation. In order to minimize the total execution time of tasks, the mechanism first divides the level of tasks based on their precedence constraints and computes expected finish time of each task by assuming that there are enough users for all the tasks. Since the total execution time of the tasks is bounded by the critical task $t_{c}$ with the maximum expected finish time, the platform should first allocate a task to a user which can minimize the expected finish time of $t_{c}$. Based on this principle, the algorithm constructs an allocation priority sequence for tasks. When a user arrives, it greedily chooses the task with the highest priority in the allocation priority sequence to allocate until all the tasks have been finished. Then, a case study is given to show how to fit the proposed mechanism in the UAV systems by considering the features of the UAV system. Finally, the simulation results show that the proposed mechanisms are efficient for crowdsensing systems with task precedence constraints. The main contributions of this work are listed as follows:

- An efficient task allocation algorithm for tasks with precedence constraints is designed. As far as we know, this is the first work which considers the precedence constraints of tasks and the proposed algorithm can minimize the total execution time of all the tasks.
- A case study is given to show how to fit the proposed mechanism in the UAV system, by considering the features of UAV task allocation problem.
- Extensive simulations are conducted to evaluate the performance of the proposed algorithm, and the results show that the proposed algorithm has good approximate optimal ratios under different parameter settings.

The remainder of the paper is organized as follows. The description of the system model is presented in Section 2. Then, the details of the proposed approximation algorithm are given in Section 3. Next, a case study is given to show how to fit the proposed mechanism in the UAV system in Section 4. Afterward, a variety of simulations are conducted to evaluate the mechanism in Section 5. Lastly, the conclusion of the whole work is presented in Section 6.

## 2. Preliminaries

In this section, the system model is first introduced in Section 2.1, and then, the formal formulation of the task allocation problem is in Section 2.2. After that, the NP-hardness of the studied problem is proved in Section 2.3.

### 2.1. System Model

**Figure 1:**
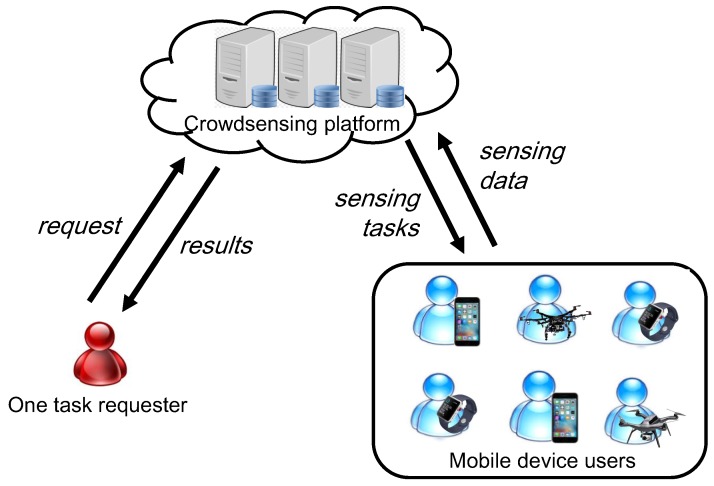
The structure of the crowdsensing system.

The crowdsensing system studied in this work is shown in Figure 1, which including a crowdsensing platform, a task requester and a set of mobile device users $U=\{u_{1},u_{2},\ldots ,u_{n}\}$. At the beginning of the task allocation, the requester will submit a set of tasks, which is denoted as $T=\{t_{1},t_{2},t_{3},\ldots ,t_{m}\}$, to the crowdsensing platform. Each task $t_{j}\in T$ can be presented as $t_{j}=\left\{C_{j},h_{j},l_{j},D_{j}\right\}$, where $C_{j}$ denotes the conditional task set of $t_{j}$, $h_{j}$ is the expected performing time of task $t_{j}$, $l_{j}$ is the location of task $t_{j}$, $D_{j}$ is the description of $t_{j}$. Due to the precedence constraints among tasks, only when all the tasks in the conditional task set $C_{j}$ have been finished, can the task $t_{j}$ be assigned to a user to perform. However, if the conditional task set $C_{j}=\phi$ (i.e., the task $t_{j}$ has no conditional task), the task $t_{j}$ can be allocated by the platform at any time.

After receiving the task request from the requester, the platform will publish all the description of tasks to the users. The users arrive online, and each available user $u_{i}$ will submit a set of interested tasks $T_{i}$ to the platform. Based on the requirement of tasks and users, the platform will allocate the tasks to users one by one. Assume that each task only needs to be performed by one user, and the platform will allocate no more than one task to each user at each round. After finishing the allocated task, the platform will add the still available users to the waiting list, and treat it as a new arrival user. Use $u_{i}=\left\{T_{i},{PH}_{i}\right\}$ to present user $u_{i}$, where $PH_{i}$ is the expected execution time set of $u_{i}$, and each ${ph}_{i,j}\in PH_{i}$ is the expected execution time of $u_{i}$ for performing task $t_{j}$.

In the UAV crowdsensing system, the task requester is the carrier, and each user is a UAV. The flight duration from the location of one task to another for different UAVs may be varied, which is an essential factor for the expected execution time of UAVs. Consider a UAV $u_{i}$ has finished task $t_{j}$ at the location $l_{j}$ and is assigned to perform next task $t_{k}$ at the location $l_{k}$. The travel duration of $u_{i}$ can be presented as ${td}_{i,k}=TD\left(u_{i},l_{j},l_{k}\right)$, where $TD(u_{i},l_{j},l_{k})$ is a function to calculate how long it generally takes for $u_{i}$ to fly from $l_{j}$ to $l_{k}$. Furthermore, if a UAV $u_{i}$ is going to finish its first task $t_{k}$, the travel duration starts from its initial location $l_{i}$ and it is denoted as ${td}_{i,k}=TD\left(u_{i},\phi ,l_{k}\right)=TD\left(u_{i},l_{i},l_{k}\right)$. Notice that $h_{k}$ is the expected performing time of task $t_{k}$. Then, ${ph}_{i,k}$ is mainly decided by ${td}_{i,k}$ and $h_{k}$ in the UAV system.

### 2.2. Problem Formulation

The goal of this work is to minimize the total execution time of the tasks with the precedence constraints.

For facilitate reading, we summarize some symbols that are used in this paper in Table 1.

Let $y_{j}=\left\{0,1\right\}$ represent whether the task $t_{j}$ is finished. If the task $t_{j}$ is a finished task, $y_{j}=1$, otherwise, $y_{j}=0$. Use $a_{j}=\left\{0,1\right\}$ to denote if the task $t_{j}$ is permitted to be allocated, and it has $a_{j}=\prod_{t_{k}\in C_{j}}y_{k}$. If task $t_{j}$ satisfies the constraint $a_{j}=1$, $t_{j}$ can be allocated to a user. Further use $x_{i,j}=\left\{0,1\right\}$ to indicate whether the platform allocates task $t_{j}$ to user $u_{i}$. If $t_{j}$ is assigned to the user $u_{i}$, $x_{i,j}=1$, otherwise, $x_{i,j}=0$. Suppose $s_{j}$ is the expected beginning time of task $t_{j}$, (i.e., the earliest time that the platform can allocate task $t_{j}$ to a user). Obviously, each $s_{j}$ should satisfy that $s_{j}\geq \max_{t_{k}\in C_{j}}\left(s_{k}+\sum_{u_{i}\in U}ph_{i,k}x_{i,k}\right)$. Define the total time that the users finish the tasks in the task set as the execution time of a task set. Then, the goal of this work is to minimize the execution time of the task set T.

**Definition 1** (The Studied Task Allocation Problem(STAP))**.** *The studied crowdsensing task allocation problem can be defined as follows:*

$$\begin{array}{c} \min\;\max_{t_{j}\in T}\left(s_{j}+{ph}_{i,j}x_{i,j}\right)\\ s.t.\;s_{j}\geq \max_{t_{k}\in C_{j}}(s_{k}+\sum_{u_{i}\in U}ph_{i,k}x_{i,k})\\ \ldots \end{array}$$

The first constraint shows that the studied allocation mechanism should satisfy the precedence constraints of tasks. Therefore, $t_{j}$ can be performed only when all the tasks in its conditional task set have been finished.

### 2.3. Analysis of the NP-Hardness

In the following, it will prove that the studied task allocation problem can be reduced to the multiprocessor scheduling problem, which is a well-known NP-hard problem [46]. The description of the multiprocessor scheduling problem is as follows: given a set of jobs and *m* processors, the goal of the multiprocessor scheduling problem is to find the minimum possible time required to schedule all jobs in the job set on *m* processors such that there is none overlap, where each job has a fixed processing time.

**Theorem** **1.** *The studied task allocation problem (STAP) is NP-hard.*

**Proof.**  Consider a simple case of the studied problem, where there is no precedence constraint among tasks, and each user is interested in all the tasks. Then, the task set in this problem can be viewed as the job set in the multiprocessor scheduling problem, and the users in the studied problem can be viewed as the processors in the multiprocessor scheduling problem. The performing time of tasks in this problem is equal to the processing time of jobs in the multiprocessor scheduling problem. Then, the goal of the problem is equivalent to find the minimum possible time required to schedule all jobs on the processors such that there is none overlap. As is known to all, the multiprocessor scheduling problem is NP-hard. Therefore, the problem studied in this work is also NP-hard, which finished the proof. □

## 3. Algorithm Design

It has been proved that the studied task allocation problem (STAP) is NP-hard, which means an approximation mechanism with polynomial-time is demanded to solve it. The proposed mechanism includes four steps, which are task level division, final task set construction, allocation priority sequence construction, and task allocation. In the first step, the levels of tasks are divided based on their conditional task sets. Define a task that is not in any conditional task set of other tasks as a final task. The total execution time of all the tasks is determined by the finishing time of final tasks. Thus, the second step of the mechanism is the construction of the final task set. Next, the mechanism achieves its design goal by sorting the tasks in descending order based on their expected finishing time. Finally, the tasks are allocated to users according to the allocation priority sequence constructed in the third step.

### 3.1. Task Level Division

The level in the algorithm is used to denote the precedence constraints among tasks. In the studied model, tasks can be allocated to users only when all the tasks in their conditional task set have been finished. Thus, the level of all the tasks in $C_{j}$ should be less than the level of $t_{j}$. Symbol $L_{j}$ is used to denote the level of $t_{j}$. Obviously, each task $t_{j}$ with $C_{j}=\phi$ is in the lowest level ( i.e., $L_{j}=1$ ). The details of how to divide the level of tasks are as shown in Algorithm 1.

**Algorithm 1** task level division**Require:**  the task set T **Ensure:**  $L={\left\{L_{j}\right\}}_{t_{j}\in T}$ 1:Set $T^{\prime }=T$;2:**for** each $t_{j}$ in T **do**3: Set $C_{j}^{\prime }=C_{j}$;4:Set k=1;5:**while**$T^{\prime }\neq \phi$**do**6: **for** each task $t_{j}\in T^{\prime }$ **do**7:  **if** $C_{j}^{\prime }=\phi$ **then**8:   Set $L_{j}=k$;9: **for** each task $t_{j}\in T^{\prime }$ **do**10:  **for** each task $t_{q}\in C_{j}^{\prime }$ **do**11:   **if** $L_{q}=k$ **then**12:    Delete $t_{q}$ from set $C_{j}^{\prime }$;13:  **if** $L_{j}=k$ **then**14:   Delete $t_{j}$ from set $T^{\prime }$;15: k++;16:**return**$L={\left\{L_{j}\right\}}_{t_{j}\in T}$;

In Algorithm 1, it first makes a copy for each conditional task set $C_{j}$, which is denoted as $C_{j}^{\prime }$. Initially the current task level k=1. The task level division algorithm runs in an iterative way. In each iteration, it scans all the tasks in temporary task set $T^{\prime }$. When $t_{j}$ is scanned, it will check whether the temporary conditional set $C_{j}^{\prime }=\phi$ or not. If $C_{j}^{\prime }=\phi$, set the task level of $t_{j}$ equal to *k* (i.e., set $L_{j}=k$). After all the tasks in $T^{\prime }$ have been scanned, it will delete the tasks with level *k* from the temporary conditional set of other tasks and delete $t_{j}$ from the temporary task set $T^{\prime }$. Finally, the algorithm sets k=k+1, and starts the next iteration until the task set $T^{\prime }=\phi$.

**Figure 2:**
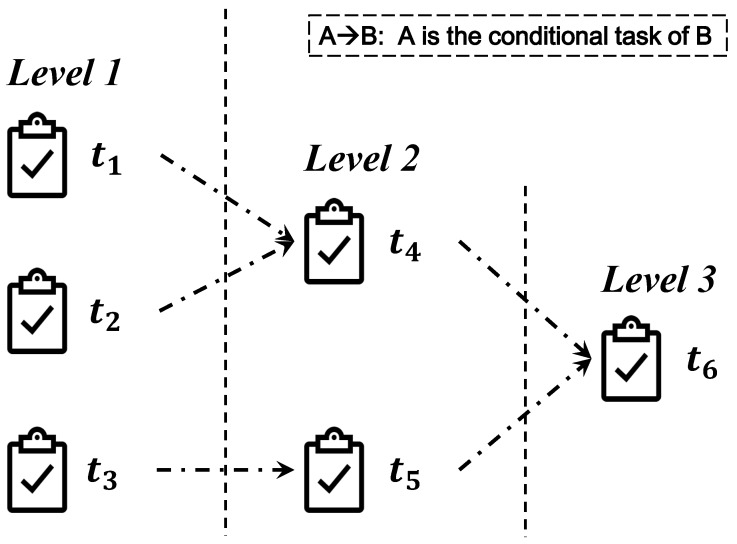
The instance for Algorithm 1.

The following instance is given to express the algorithm more clearly. Suppose the task set $T=\{t_{1},t_{2},t_{3},\ldots ,t_{6}\}$ in Figure 2.

According to Algorithm 1, the level of each task in the task set T is gotten. Apparently, tasks are divided into three levels. $t_{1},t_{2},t_{3}$ are in the first level, $t_{4},t_{5}$ are in the second level and $t_{6}$ is in the last level.

### 3.2. Final Task Set Construction

**Definition** **2.** *Define the tasks that don’t exist in any other tasks’ conditional task set as final tasks. There exists at least one final task $t_{j}$ in T. If $t_{j}$ is a final task, then $t_{j}\notin C_{k}$ stands for all the $t_{k}\in T$.*

Since the total execution time of the tasks is bounded by final tasks, construction of the final task set is performed before sorting the allocation priority of tasks. In Algorithm 2, all the tasks in T are firstly assumed as final tasks. Then, they are checked one after another. When task $t_{j}$ is checked, it will be deleted from the final task set F if it exists in the conditional task set of at least one task. The details are as shown in Algorithm 2.

**Algorithm 2** Final task set construction**Require:**  the task set T **Ensure:**  the final task set F 1: Set F=T; 2: **for** each task $t_{j}\in F$
**do** 3:  **for** each task $t_{k}\in T$
**do** 4:   **for** each task $t_{q}\in C_{k}$
**do** 5:    **if**
$t_{j}=t_{q}$
**then** 6:     Delete task $t_{j}$ from the final task set F; 7: **return** the final task set F;

Consider the instance in Section 3.1. There is no conditional task set $C_{k}\left(k\in \left[1,6\right]\right)$ in this example contains task $t_{6}$. Thus, the constructed final task set $F=\{t_{6}\}$. Obviously, all the tasks in T should be done when all the final tasks in F have been finished, and it is the feature of the final task.

### 3.3. Allocation Priority Sequence Construction

The optimization objective is bounded by the final tasks with the maximum expected finishing time. Thus, all the expected finishing time of the final tasks should be computed when tasks are allocated to users. To achieve the designed goal, the algorithm sorts the tasks with their expected finishing time and constructs an allocation priority sequence. In the following, some important definitions are given first.

**Definition** **3.** Task Sequence*: A task sequence is sequence of tasks which satisfies the l-th task in the sequence should be in the conditional task set of the l+1-th task and this sequence ends in a final task.*

Since the tasks can only be performed one by one in the task sequence, the expected finishing time of a task sequence is equal to the expected finishing time of the final task in the sequence.

**Definition** **4.** *The Critical Task Sequence: The critical task sequence is defined as the task sequence with maximum expected finishing time.*

The allocation priority sequence construction algorithm runs in iterations. In each iteration, the critical task sequence of the task set T has to be found first, then the algorithm puts the task with the lowest level in the critical task sequence into the allocation priority sequence. The details are as shown in Algorithm 3.

Suppose $f_{j}$ is the order of task $t_{j}$ in the allocation priority sequence. In each iteration, the algorithm aims to find a task sequence and its value is greater than any other task sequences. Note that the value of a task sequence is equal to the expected finishing time of the final task in the sequence. To get the expected finishing time of the final tasks, the expected finishing time of the tasks in their conditional task set should be calculated. Thus, Algorithm 3 first computes the expected finishing time of each task in T.

Let $h_{j}^{e}$ be the expected finishing time of task $t_{j}$. Note that the expected execution time of different users for the same task may be different. However, users arrive online in this work. Thus, it hardly to get the expected execution time of tasks before allocating. In order to solve this problem, the mechanism assumes the expected execution time of task $t_{j}$ is $h_{j}$ in this step. Then, it has $h_{j}^{e}=h_{j}$ when $L_{j}=1$. When $L_{j}\geq 2$, $h_{j}^{e}=h_{j}+\max{\left\{h_{p}^{e}\right\}}_{t_{p}\in C_{j}}$. Notice that the algorithm computes the expected finishing time of tasks from low level to high level, and the levels of tasks in $C_{j}$ are lower than $t_{j}$. Thus, ${\left\{h_{p}^{e}\right\}}_{t_{p}\in C_{j}}$ are known when it computes $h_{j}^{e}$. $c_{j}^{t}$ is used to record the task with maximum expected finishing time among tasks in $C_{j}$, which can help to construct the critical task sequence.

After computing the expected finishing time of all the final tasks at the start of the algorithm, the mechanism begins to construct the priority sequence in iterations. As the expected finishing time of all the final tasks is calculated, the task $t_{q}$ with maximum expected finishing time can be found, and construction of the critical task sequence of task $t_{q}$ is available with the help of recorded $c_{j}^{t}$. Suppose $t_{p}$ is the task with lowest level in the critical task sequence of final task $t_{q}$, $t_{p}$ is put into the allocation priority sequence by setting $f_{p}=l$. Then, $t_{p}$ is deleted from task set T. If $t_{p}$ is a final task, $t_{p}$ should also be deleted from the final task set F. Afterward, the algorithm finds tasks that are directly or indirectly related with the deleted $t_{p}$ and computes their expected finishing time. Finally, the next iteration begins until T=ϕ.

**Figure 3:**
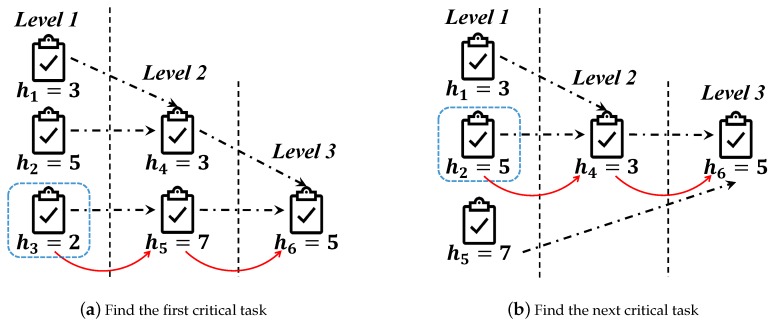
The instance for Algorithm 3.

Based on the information of tasks in Figure 2, the allocation priority sequence is gotten by continuously finding a new critical task sequence for a changed task set T. Figure 3a is the situation of task set T when the first task in allocation priority sequence has been found, and Figure 3b corresponds to the second task in the sequence. In Figure 3a, the task sequence $<t_{3},t_{5},t_{6}>$ is the critical task sequence, then, the task $t_{3}$ is the first task in allocation priority sequence. In Figure 3b, the task sequence $<t_{2},t_{4},t_{6}>$ is the critical task sequence in the changed task set $T-\{t_{3}\}$, and the task $t_{2}$ is the second task in the allocation priority sequence. Furthermore, in order to find the third task in the allocation priority sequence, the mechanism ought to find the critical task sequence in the changed task set $T-\{t_{2},t_{3}\}$.

**Algorithm 3** Allocation priority sequence construction**Require:**  the task set T, the final task set F,  the refresh task sequence R; **Ensure:**  the allocation priority sequence ${\left\{f_{j}\right\}}_{t_{j}\in T}$; 1:**for**k=1 to $\max{\left\{L_{j}\right\}}_{t_{j}\in T}$ **do**2: **for** each task in T **do**3:  **if** $L_{j}=k$ **then**4:   Set $c_{j}^{t}$ is the task with the maximum expected finishing time among all the tasks in $C_{j}$.5:   Compute the expected finishing time $h_{j}^{e}$;6:Set l=1;7:**while**T≠ϕ**do**8: Find the final task $t_{q}\in F$ with maximum expected finishing time;9: Construct the critical task sequence of task $t_{q}$;10: Set $f_{p}=l$;11: Find the task $t_{p}$ with the lowest level in the critical task sequence of task $t_{q}$;12: Delete $t_{p}$ from T;13: **if** $t_{p}\in F$ **then**14:  Delete $t_{p}$ from F;15: Gather all the rest tasks that have priority relationship with $t_{p}$ in the sequence R;16: **for** each task $t_{j}$ in R **do**17:  Set $c_{j}^{t}$ is the task with the maximum expected finishing time among all the tasks in $C_{j}$.18:  Compute the expected finishing time $h_{j}^{e}$;19:  Add all the rest tasks that have priority relationship with $t_{j}$ to the back of sequence R;20: Set l=l+1;

### 3.4. Task Allocation

After constructing the allocation priority sequence, the platform allocates tasks to users according to the order in the constructed allocation priority sequence. The proposed task allocation mechanism runs iteratively. In each iteration, the platform greedily allocates one task to a user, which will minimize the expected total execution time of all tasks. The details are as follows:

Step 1: Sort the tasks in T according to the allocation priority sequence.

Step 2: In each iteration, the task with the highest priority in the sorted task list should be found first, and its conditional task set is ϕ. Assume this task is $t_{j}$. Next, compute the expected execution time of all users interested in task $t_{j}$. Then, the user $u_{i}$ who interested in $t_{j}$ with the minimum $pt_{i,j}$ can be found. The platform allocates $t_{j}$ to $u_{i}$ in this iteration. After that, the platform deletes $u_{i}$ from the available user set and deletes $t_{j}$ from the task set T. Then, the next iteration begins until the task set T=ϕ. In the case of there is no task can be allocated to users, and all the tasks and users remain in T and W should wait for new arrive users or some of the allocated tasks finished.

Step 3: When an allocated task $t_{j}$ has finished by $u_{i}$, the platform deletes $t_{j}$ from all the conditional task sets that include $t_{j}$. If $u_{i}$ is still available, the platform adds $u_{i}$ into the available user set, and views it as a new arrival user. Then, run step 2.

Note that the expected total execution time will be minimized if the tasks are performed in the order of the constructed allocation priority sequence. The proposed allocation mechanism greedily choose the task with the highest priority to allocate in each iteration, which means the mechanism can achieve a near-optimal total execution time.

## 4. A Case Study: Task Allocation Mechanism for UAV System

This section is to show how to fit the proposed task allocation mechanism in the UAV system.

Consider a Crowdsensing based UAV system, there exists a carrier, a control platform and a group of UAVs embedded with different kinds of sensing devices. At the beginning of each round allocation, the carrier first submits the tasks to the platform. The platform has an available UAV list, and each available UAV submits a set of tasks that it can perform to the platform. Then, the platform runs Algorithms 1 and 2 to compute the level of each task and construct the final task set.

As is introduced in Section 2.1, the expected execution time of a UAV for task $t_{j}$ is mainly decided by the flight duration from the current location to $L_{j}$ and the expected performing time of $t_{j}$ in the UAV system. Although the flight time of different UAV may be varied, the expected performing time of different UAVs is similar for a fixed task. Therefore, the expected performing time of $t_{j}$ can be assumed to equal to $h_{j}$ for all the UAVs that have ability to perform $t_{j}$. In the step of constructing the allocation priority sequence, set $h_{j}^{e}=h_{j}+\max{\left\{h_{p}^{e}\right\}}_{t_{p}\in C_{j}}$. By running Algorithm 3, the platform can get the allocation priority sequence of tasks.

Then, the platform adds the UAVs in a waiting list, and allocates the tasks to them based on the allocation priority sequence. In each iteration of allocation, the platform allocates a task to a user that can minimize the total execution time of all the tasks, i.e., allocates a task with the lowest level in the critical sequence to the user with minimal expected execution time. Based on the proposed mechanism of constructing the allocation priority sequence, the task with the lowest level in the critical sequence is the task with the highest priority in the sequence. Assume $t_{j}$ is this specific task. The expected execution time of a UAV $u_{i}$ for performing $t_{j}$ is ${ph}_{i,j}={td}_{i,j}+h_{j}$.

When a UAV has finished an allocated task $t_{j}$, it will be added to the waiting list again. Moreover, the platform will delete $t_{j}$ from all the conditional task sets that include $t_{j}$. This process goes on, until all the tasks have been finished.

## 5. Simulation

In this section, the settings of all the parameters are introduced first, and then extensive simulations are conducted to evaluate the proposed mechanisms.

### 5.1. Simulation Setting

A task is not always allocated immediately once it is available. More in details, when a task is available to be allocated, it might wait some time before being allocated. Furthermore, the total execution time of all the tasks is not likely to equal the theoretically optimal value of the allocation for the task set. Thus, the approximate optimal ratio is related to the performance of the algorithm in simulations.

**Definition** **5.** *The parameter Alg denotes the execution time of task set T under the allocation of an algorithm, and Opt represents the theoretically optimal value of the allocation for the task set T. Then, the* approximate optimal ratio*: $\eta =\frac{Alg}{Opt}$.*

**Figure 4:**
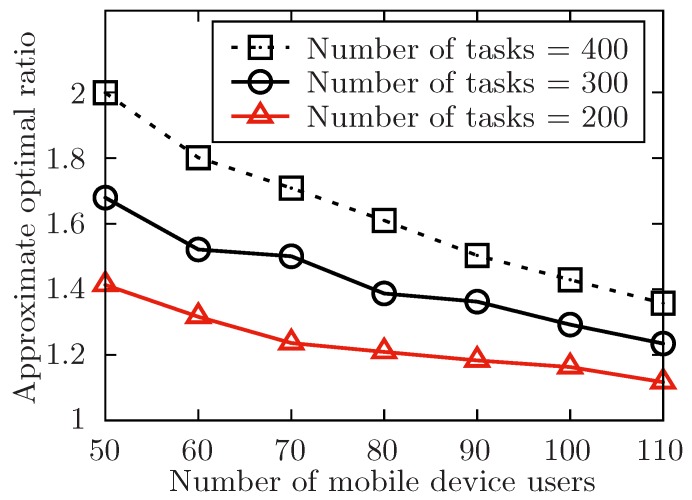
The approximate optimal ratio of the proposed algorithm vs. different *n* when m=200,300,400.

**Figure 5:**
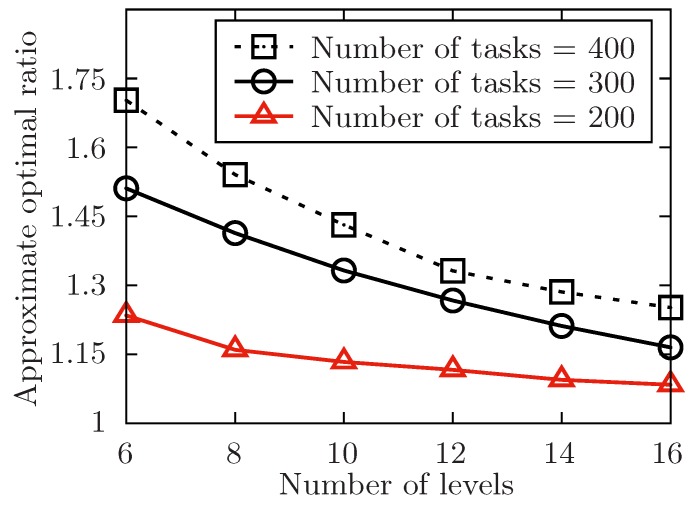
The approximate optimal ratio of the proposed algorithm vs. different *l* when m=200,300,400.

**Figure 6:**
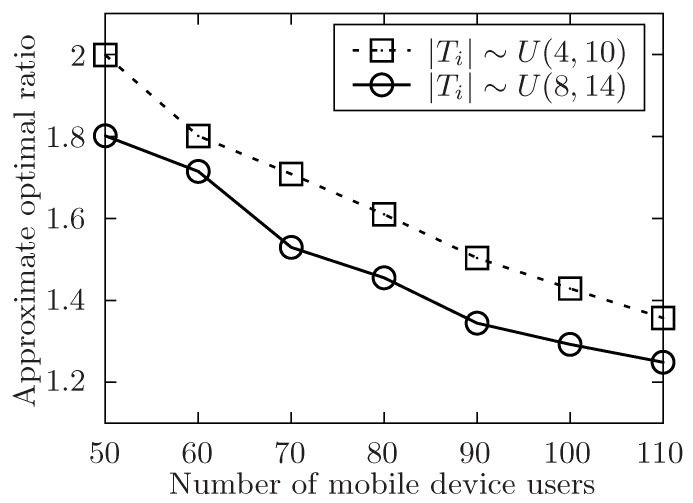
The approximate optimal ratio of the proposed algorithm vs. different *n* when $|T_{i}|∼U\left(4,10\right)$ or U(8,14).

**Figure 7:**
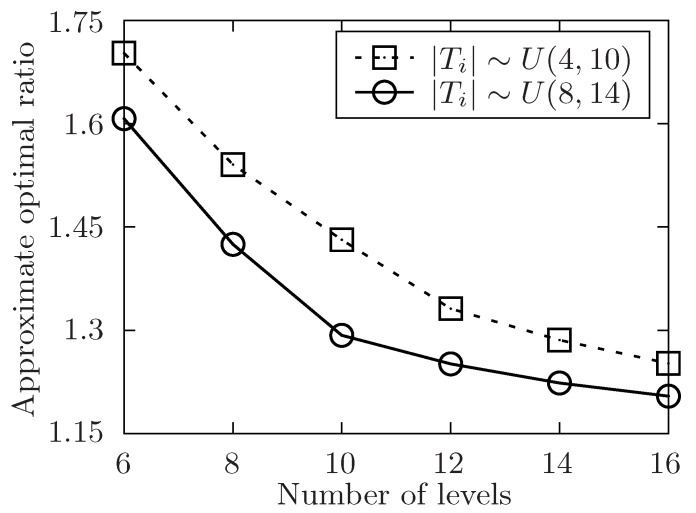
The approximate optimal ratio of the proposed algorithm vs. different *l* when $|T_{i}|∼U\left(4,10\right)$ or U(8,14).

**Figure 8:**
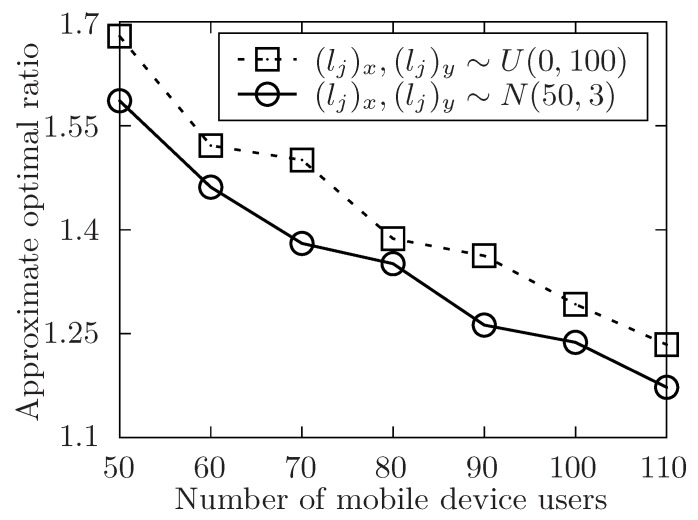
The approximate optimal ratio of the proposed algorithm vs. different *n* when ${\left(l_{j}\right)}_{x},{\left(l_{j}\right)}_{y}∼U\left(0,100\right)$ or N(50,3).

**Figure 9:**
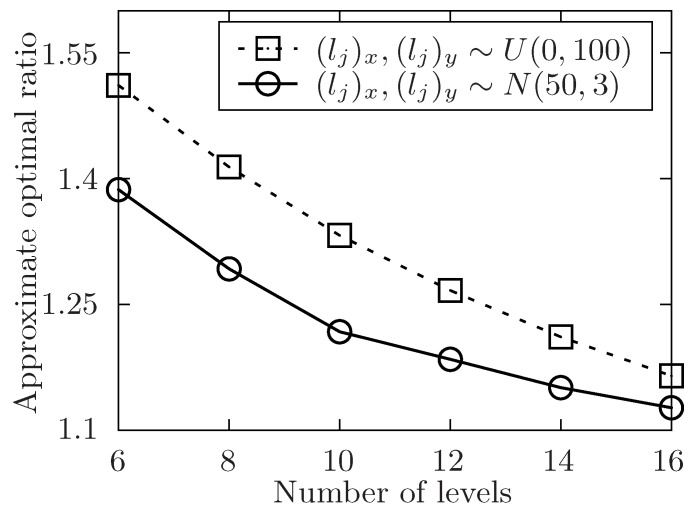
The approximate optimal ratio of the proposed algorithm vs. different *l* when ${\left(l_{j}\right)}_{x},{\left(l_{j}\right)}_{y}∼U\left(0,100\right)$ or N(50,3).

**Figure 10:**
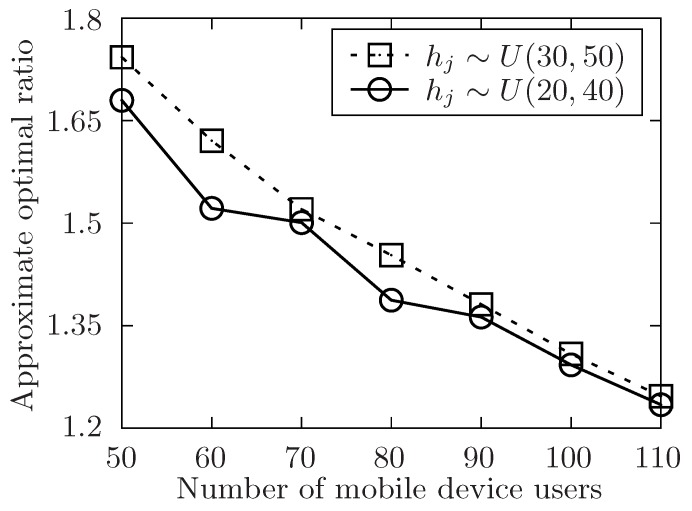
The approximate optimal ratio of the proposed algorithm vs. different *n* when $h_{j}∼U\left(20,40\right)$ or U(30,50).

**Figure 11:**
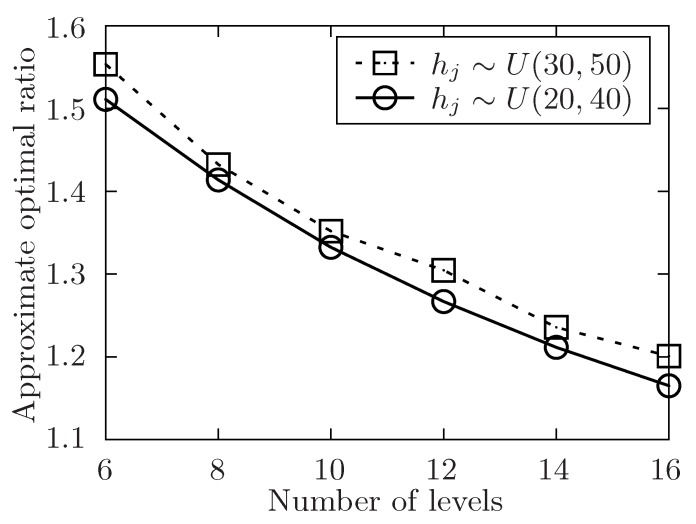
The approximate optimal ratio of the proposed algorithm vs. different *l* when $h_{j}∼U\left(20,40\right)$ or U(30,50).

The setup of the simulation is as follows. In order to show the performance of the proposed algorithm, it varies the number of the released tasks, the number of the involved mobile device users and the level of the tasks set T with the symbol of *m*, *n*, *l*. Besides, m=|T|, n=|U| and $l=\max{\left\{L_{j}\right\}}_{t_{j}\in T}$. The number of total tasks in each level is uniformly distributed in [m/l−5,m/l+5]. For any task $t_{j}$ in task set T, it has attributes of expected performing time $h_{j}$, conditional task set $C_{j}$, and its location $l_{j}$. The size of $C_{j}$ is always distributed in [1,4] at random. In Figure 4, Figure 5, Figure 6, Figure 7, Figure 8 and Figure 9, the parameter $h_{j}$ appears to follow the uniform distribution in U[20,40]. And in Figure 10 and Figure 11 simulations, the parameter $h_{j}$ can also be U(30,50) uniformly distributed. Each user $u_{i}$ submits a set of tasks $T_{i}$ that he is interested in performing, and the size of $T_{i}$ is randomly generated in [4,10] or [8,14] in different experiments. Furthermore, in Figure 4, Figure 5, Figure 8, Figure 9, Figure 10 and Figure 11, the parameter $|T_{i}|$ always follows U[4,10] uniform distribution. And in Figure 6 and Figure 7 simulations, the parameter $|T_{i}|$ is also set as U(8,14) uniformly distributed. What’s more, to show that the proposed mechanism is available to be applied to the UAV system, settings about both tasks and users’ location are also made. In Figure 4, Figure 5, Figure 6 and Figure 7, Figure 10 and Figure 11, both the locations of tasks and users are uniformly distributed in U(0,100). And in Figure 8 and Figure 9, the location can also be normally distributed in N(50,3). If a user $u_{i}$ is assigned to finish the task $t_{j}$, calculate the Euclidean distance of the user and task, and relate it to the travel duration of the user $u_{i}$ for the task $t_{j}$. After that, the requested performing time of a user $u_{i}$ to perform task $t_{j}$ is determined.

In each case of <m,n,l>, the simulation generates 2000 instances and takes the average value of them. The average value is the outcome of the case finally. The settings of all cases and the outcomes of simulations are shown in Table 2 and Table 3.

### 5.2. Simulation Results

In Figure 4, Figure 6, Figure 8 and Figure 10, the performance of the proposed algorithm is validated by changing the number of released tasks in different parameter settings. Regardless of other parameters’ settings, it is obvious that when the number of involved users increases, the approximate optimal ratio decreases. As the theoretically optimal value of the allocation for the task set T is only in connection with the structure of the task set T, and the theoretically optimal value is fixed no matter how involved users change. When the number of involved users increases, the time of task’s waiting to be allocated is likely to decrease, which would make the execution time of the task set T decrease. Then, the approximate optimal ratio decreases. Therefore, the approximate optimal ratio decreases as the number of involved users increases.

In Figure 5, Figure 7, Figure 9 and Figure 11, the performance of the proposed algorithm is shown by changing the level of the tasks set in different environment setting. Apparently, the approximate optimal ratio will decrease if the levels of the task set increases. It is because more levels of task set make the number of tasks in each level less, the tasks are more likely to be allocated once they are available, and the waiting time of tasks in task set T is likely to decrease, which would make the execution time of the task set T closer to the theoretically optimal value of the allocation for the task set. Thus, the approximate optimal ratio decreases as the level of task set increases.

In Figure 4 and Figure 5, the performance of the algorithm is illustrated by changing the number of released tasks. In both two settings, the number of released tasks *m* ranges in {200,300,400}. In Figure 4, it changes the value of involved users *n* from 50 to 110 while the level of task set *l* is set to be 6. And in Figure 5, it changes the level of the task set *l* from 6 to 16 while the number of involved users *n* is set to be 70. Both two simulations show that the approximate optimal ratio increases with the increasing number of released tasks. As the theoretically optimal value of the allocation for the task set T is only in connection with the structure of the task set T, and the theoretically optimal value is fixed no matter how the number of released tasks changes. When the number of released tasks increases, the time of each task waiting to be allocated is likely to increase, and the execution time of the released task set is also to increase, which would increase the execution time of the task set T.

In Figure 6 and Figure 7, the size of involved users’ interested task set $T_{i}$ is in different range. In these two simulations, the performance of the algorithm is validated by changing the size of user’s submitted task set. Furthermore, the size of user’s submitted task set is uniformly distributed in U(4,10) or U(8,14). In both two settings, the number of released task *m* is fixed in 400. In Figure 6, the number of involved users *n* is changed from 50 to 110 while the level of task set *l* is set to be 6. And in Figure 5, it changes the level of task set *l* from 6 to 16 while the number of involved users *n* is set to be 70. Both two simulations show that the approximate optimal ratio decreases with the increasing number of user’s submitted tasks. As the theoretically optimal value is fixed no matter how the number of released tasks changes. When the number of submitted tasks increases, and there exists some users in the available user list, the time of each task’s waiting to be allocated is more likely to decrease, and the execution time of the released task set is also to decrease, which would make the execution time of the task set T decrease.

In Figure 8 and Figure 9, the performance of the algorithm is shown by changing the area of region that the released tasks and involved users locate in. In both two settings, the number of released task *m* is fixed to 300. In Figure 8, the number of involved users *n* ranges from 50 to 110 while the level of task set *l* is set to be 6. And in Figure 9, the level of task set *l* ranges from 6 to 16 while the number of involved users *n* is set to be 70. Both two simulations show that the approximate optimal ratio increases with the increasing of the area of regions. It is because when the area of region increases, the performing time of released tasks increases, then the time of each task waiting to be allocated is likely to increase, and the execution time of the released task set is also to increase, which would increase the execution time of the task set T.

In Figure 10 and Figure 11, the performance of the algorithm is illustrated by changing the expected performing time of released tasks. In both two settings, the number of released task *m* is fixed to 300. In Figure 10, the number of involved users *n* is changed from 50 to 110 while the level of task set *l* is set to be 6. And in Figure 11, it changes the level of task set *l* from 6 to 16 while the value of involved users *n* is set to be 70. Both two simulations show that the approximate optimal ratio increases with the increasing expected performing time of released tasks. When expected performing time of released tasks increases, the time users have to wait for each task to be allocated may increase, and the execution time of the released task set is also to increase, which would make the execution time of the task set T increase.

## 6. Conclusions

In this paper, the precedence constraints of tasks are considered, and an efficient task allocation algorithm is designed for crowdsensing systems with the goal of minimizing the total execution time of the tasks. The proposed algorithm first divides tasks into multiple levels and finds all the final tasks. Then, it constructs an allocation priority sequence according to the expected finishing time of tasks, and allocates the tasks to users based on the constructed allocation priority sequence. Finally, a case study is given to show how to fit the designed mechanism in the UAV system. The simulation results verify the efficiency of the designed mechanism.

In future work, the deadlines of tasks and the available time intervals of users will be taken into consideration when designing an efficient task allocation mechanism for tasks with precedence constraints. Moreover, the plans for designing a mobile crowdsensing based traffic congestion monitoring system and exploring real-world experimentation for the proposed task allocation mechanism also deserve to be carried out.

References

## Figures and Tables

**Table 1:** The descriptions of notations used in this paper.

Notation	Description
U	a set of mobile device users
$u_{i}$	a mobile device user *i*
$T_{i}$	interested tasks submitted by user *i*
${PH}_{i}$	the expected execution time set of user *i*
${ph}_{i,j}$	the expected execution time of user *i* for performing task *j*
T	the task set submitted by requester
$t_{j}$	a task *j*
$C_{j}$	the conditional task set of task *j*
$h_{j}$	the expected performing time of task *j*
$l_{j}$	the location of task *j*
$D_{j}$	the description of task *j*
${td}_{i,k}$	the travel duration of user *i* to the location of task *k*
$y_{j}$	binary variable to represent whether task *j* is finished
$a_{j}$	binary variable to represent if task *j* is permitted to be allocated
$x_{i,j}$	binary variable to represent whether task *j* is allocated to user *i*
$s_{j}$	the earliest time that the platform can allocate the task *j* to a user
$L_{j}$	the level of task *j*
F	the final task set
$f_{j}$	the order of task *j* in allocation priority sequence
$h_{j}^{e}$	the expected finishing time of task *j*
$c_{j}^{t}$	the recorded task with maximum expected finishing time in $C_{j}$

**Table 2:** The description of different cases.

Case	Description				Fixed Parameter
	m	$|T_{i}|$	$h_{j}$	${\left(l_{j}\right)}_{x}$ and ${\left(l_{j}\right)}_{y}$	
A	200	U(4,10)	U(20,40)	U(0,100)	l=6 or n=70
B	300	U(4,10)	U(20,40)	U(0,100)	
C	400	U(4,10)	U(20,40)	U(0,100)	
D	400	U(8,14)	U(20,40)	U(0,100)	
E	300	U(4,10)	U(30,50)	U(0,100)	
F	300	U(4,10)	U(20,40)	N(50,3)	

**Table 3:** The approximate optimal ratios under different cases.

Approximate Optimal Ratios													
**Case**	**Number of Mobile Device Users (n)**							**Number of Levels (l)**					
	**50**	**60**	**70**	**80**	**90**	**100**	**110**	**6**	**8**	**10**	**12**	**14**	**16**
A	1.413	1.316	1.236	1.209	1.183	1.163	1.117	1.234	1.160	1.133	1.116	1.094	1.084
B	1.679	1.521	1.500	1.387	1.362	1.292	1.234	1.511	1.414	1.332	1.267	1.211	1.165
C	1.999	1.801	1.708	1.610	1.503	1.429	1.357	1.702	1.541	1.431	1.331	1.286	1.252
D	1.802	1.715	1.529	1.455	1.344	1.293	1.249	1.607	1.425	1.293	1.251	1.223	1.204
E	1.743	1.620	1.520	1.453	1.381	1.308	1.246	1.553	1.432	1.352	1.305	1.235	1.201
F	1.586	1.462	1.380	1.351	1.262	1.238	1.172	1.387	1.292	1.217	1.185	1.151	1.127
